# Evaluation of epilepsy lesion visualisation enhancement in low-field MRI using image quality transfer: a preliminary investigation of clinical potential for applications in developing countries

**DOI:** 10.1007/s00234-024-03448-2

**Published:** 2024-09-06

**Authors:** Matteo Figini, Hongxiang Lin, Felice D’Arco, Godwin Ogbole, Maria Camilla Rossi-Espagnet, Olalekan Ibukun Oyinloye, Joseph Yaria, Donald Amasike Nzeh, Mojisola Omolola Atalabi, David W. Carmichael, Judith Helen Cross, Ikeoluwa Lagunju, Delmiro Fernandez-Reyes, Daniel C. Alexander

**Affiliations:** 1 Centre for Medical Image Computing, University College London, 90 High Holborn, London, WC1V 6LJ UK; 2 Computer Science, University College London, London, UK; 3 Zhejiang Lab, Hangzhou, China; 4 Radiology, Great Ormond Street Hospital for Children, London, UK; 5 Radiology, College of Medicine, University of Ibadan, Ibadan, Nigeria; 6 Functional and Interventional Neuroradiology Unit, Bambino Gesù Children’s Hospital, IRCCS Rome, Rome, Italy; 7 Radiology, University of Ilorin Teaching Hospital, Ilorin, Nigeria; 8 Neurology, University College Hospital Ibadan, Ibadan, Nigeria; 9 School of Biomedical Engineering & Imaging Sciences, King’s College London, London, UK; 10 University College London Great Ormond Street Institute of Child Health, London, UK; 11 Great Ormond Street Hospital for Children, London, UK; 12 Paediatrics, College of Medicine, University of Ibadan, Ibadan, Nigeria

**Keywords:** Low-field MRI, Deep learning, Image quality transfer, Lesion visualisation, Epilepsy, Super-resolution

## Abstract

**Purpose:**

Low-field (LF) MRI scanners are common in many Low- and middle-Income countries, but they provide images with worse spatial resolution and contrast than high-field (HF) scanners. Image Quality Transfer (IQT) is a machine learning framework to enhance images based on high-quality references that has recently adapted to LF MRI. In this study we aim to assess if it can improve lesion visualisation compared to LF MRI scans in children with epilepsy.

**Methods:**

T1-weighted, T2-weighted and FLAIR were acquired from 12 patients (5 to 18 years old, 7 males) with clinical diagnosis of intractable epilepsy on a 0.36T (LF) and a 1.5T scanner (HF). LF images were enhanced with IQT. Seven radiologists blindly evaluated the differentiation between normal grey matter (GM) and white matter (WM) and the extension and definition of epileptogenic lesions in LF, HF and IQT-enhanced images.

**Results:**

When images were evaluated independently, GM-WM differentiation scores of IQT outputs were 26% higher, 17% higher and 12% lower than LF for T1, T2 and FLAIR. Lesion definition scores were 8–34% lower than LF, but became 3% higher than LF for FLAIR and T1 when images were seen side by side. Radiologists with expertise at HF scored IQT images higher than those with expertise at LF.

**Conclusion:**

IQT generally improved the image quality assessments. Evaluation of pathology on IQT-enhanced images was affected by familiarity with HF/IQT image appearance. These preliminary results show that IQT could have an important impact on neuroradiology practice where HF MRI is not available.

**Supplementary Information:**

The online version contains supplementary material available at 10.1007/s00234-024-03448-2.

## Introduction

Low-field (LF, < 1T) Magnetic Resonance Imaging (MRI) scanners are common in most Low- and Middle-Income countries (LMICs), mainly due to infrastructure complexities and costs that prevent the use of standard high-field (HF, 1.5 or 3T) scanners. Interest in low-field MRI is also growing considerably in High-Income Countries (HICs), due to recent technical developments for portable MRI scanners [1].

LF MRI has inherently lower signal-to-noise ratio (SNR) compared to HF, and images are usually acquired at lower resolution to partially counteract this loss in signal [2]. The contrast to noise ratio (CNR) between tissues, in particular, grey matter (GM) and white matter (WM) in the brain, is also usually lower than at HF. This reduction in SNR and CNR or resolution impairs the diagnostic value of LF MRI, especially for the detection and characterisation of small and subtle lesions.

Image Quality Transfer (IQT) is a machine learning framework that aims to estimate, from a low-quality image, the image that would have been obtained from a state-of-the-art scanner on the same subject [3–5]. It has been recently adapted for the enhancement of low-field MRI, aiming both to increase the spatial resolution in the slice direction of the input LF images and to enhance the contrast between tissues, approaching that of 3T MRI [6–8]. Previous work shows preliminary results in simulated images and in a few real cases, but has not investigated the implications of such quality improvements for the radiological evaluation of MR images.

Here we report the first results of a clinical evaluation by 7 radiologists who blindly reviewed and rated the diagnostic quality of LF, HF, and IQT-enhanced LF images from 12 paediatric patients with epilepsy. We chose the application to paediatric epilepsy for this proof-of-concept study because of its relevance in LMICs and because epilepsy often presents with subtle lesions, the characterisation of which is very challenging at LF but of great importance in the context of epilepsy surgery. The purpose is to assess if IQT can improve the quality and lesion evaluation of LF structural MRI, using HF scans from the same patients as references.

## Methods

### MRI scans

Twelve paediatric patients (5 to 18 years old, 7 males and 5 females) with clinical diagnosis of intractable epilepsy were included in this study. They had MRI scans both on a LF 0.36T MRI scanner (MagSense 360, Mindray, China) and on a HF 1.5T scanner (Signa, GE Healthcare, Milwaukee, WI, USA) between June and October 2019. On both scanners, Fluid-Attenuated Inversion Recovery (FLAIR), T1-weighted (T1w) and T2-weighted (T2w) images were acquired in axial orientation with an in-plane resolution of 0.5 mm, a slice thickness of 5 mm and a slice gap of 1 mm, to reflect the routine acquisition protocols on the LF scanner; 3D T1w images were also acquired at 1.5T as anatomical reference.

### Image preprocessing

All images underwent brain extraction using the unified segmentation algorithm [9] in Statistical Parametric Mapping 12 (SPM12, Functional Imaging Laboratory, University College London, London, UK) and correction of bias field artifacts with the N4 algorithm [10] in Advanced Normalization Tools (ANTs); T1w LF images were also corrected for cross-talk artifacts in Matlab R2023a (MathWorks, Natick, USA) by histogram equalisation of each slice with one central slice of the same volume. All the pre-processed images were carefully reviewed to make sure that no artifacts or unwanted changes were introduced.

### Image quality transfer

An IQT model was trained on pairs of patches of high-resolution HF images from either the WU-Minn Human Connectome Project [11] or the Leipzig Study for Mind-Body-Emotion Interactions [12] and synthetic LF images obtained by blurring, downsampling and changing the contrast of the corresponding HF images to match the resolution and the typical contrast and SNR of real LF images. The backbone of the IQT model used the anisotropic (ANISO) U-Net [6], allowing super resolution in a single direction by anisotropic downsampling and deeper layers in the concatenation paths of the vanilla 3D U-Net. The loss function was the average voxel-wise mean square error over all training patch pairs; it used the ADAM optimiser, an initial learning rate of 10^− 3^ and a decay of 10^− 6^ and ran for 100 epochs. See [8] for more details; the python code is freely available at https://github.com/hongxiangharry/Stochastic-IQT.

The trained model was applied to the axial LF images to obtain IQT-enhanced images. The HF images acquired in this study were not used as input of the IQT algorithm but only as a reference of HF quality for evaluations the following evaluations.

### Radiological evaluation

All the images were anonymized by removing both patient’s data and scanner information, so that readers were blinded to the field strength of the images they were viewing. The images were randomly assigned to 7 reviewers, 2 with specialist expertise in paediatric neuroimaging and more than 5 years of experience, mainly working on high-field MR images, and 5 with general radiology training and experience mainly on low-field MR images.

We performed two experiments in which the reviewers were asked to evaluate both the differentiation between normal-appearing grey (GM) and white matter (WM) and the capability to evaluate the extension and definition of the epileptogenic lesions (when present) with a score of 1 (not visible), 2 (subtle), 3 (clear) or 4 (very clear). In a first experiment, each reviewer was presented with one set of images (FLAIR, T1w and T2w) for each patient, randomly taken from the HF, LF or IQT-enhanced LF acquisition, to assess the diagnostic confidence in each image type presented individually. In a second experiment all the 3 sets of images (HF, LF and IQT-enhanced LF, each of them including FLAIR, T1w and T2w images) were presented side by side in a random order for each patient, to directly compare the contrast and lesion conspicuity in the images. For each image type and sequence, the scores were averaged across patients and reviewers, excluding any missing data (in particular lesion scores in lesion-negative scans). For lesions, the diagnosis by the most experienced neuroradiologist on the HF scans was considered as the reference.

The Wilcoxon signed rank test was applied to assess the pairwise differences in each score between IQT and either LF or HF images, separately for T1w, T2w and FLAIR.

## Results

Patients’ demographic information and the reference diagnosis from HF images is reported in Table 1. Three out of the 12 patients were considered non-lesional at HF, while the remaining 9 had lesions. As only one subtle lesion was found in this cohort and most abnormalities could be seen on all images by all reviewers, we did not perform an accuracy analysis on the number of detected lesions but only evaluated their extension and definition as described above, as well as the GM-WM differentiation.

**Table 1:** Study participants characteristics. For each patient the four columns include the progressive number, age at MRI (years), sex (F = Female, M = Male) and the diagnosis made by the most experienced neuroradiologist from HF images

Patient number	Sex	Age at LF MRI	Time between LF and HF MRI	Diagnosis on HF images
01	F	15	33 days	Left MCA ischemia
02	M	18	33 days	Non-lesional
03	M	10	43 days	Tuberous sclerosis (sub ependymal nodules and tubers)
04	M	9	43 days	Infarct
05	M	6	22 days	Occipital cortico-subcortical lesions (neonatal hypoglycemia)
06	F	7	22 days	Non-lesional (possibly therapy-related atrophy)
07	M	6	1 day	Callosal agenesis and ventriculomegaly
08	F	5	15 days	Hypoxic ischemic injury
09	F	12	1 day	Pilocytic astrocytoma
10	M	10	15 days	Left temporal pole focal cortical dysplasia
11	M	13	15 days	Left MCA ischemia
12	F	12	1 day	Non-lesional (possibly therapy-related atrophy)

**Fig. 1:**
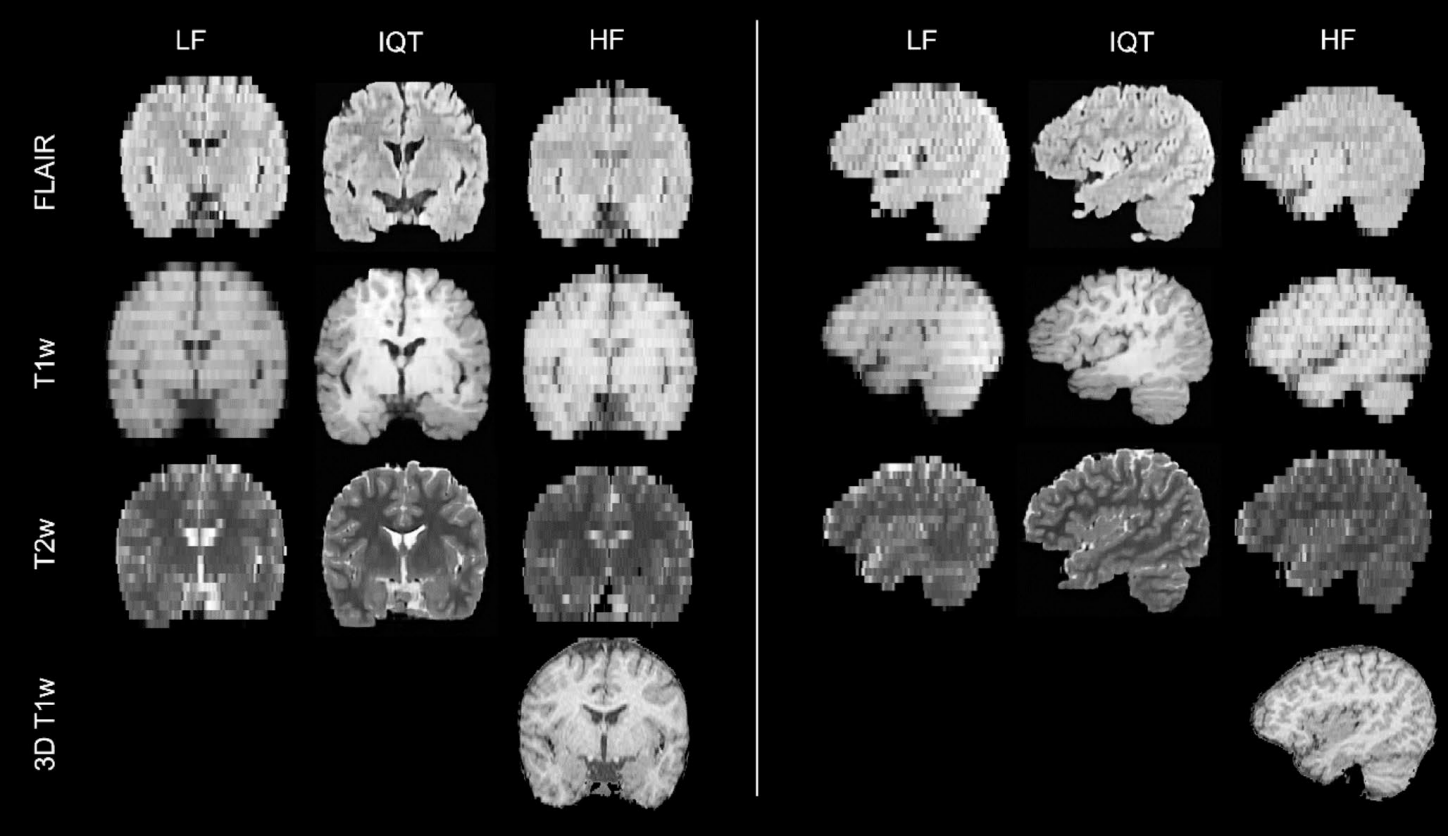
Representative coronal (left) and sagittal (right) reformatted images from a 13-year-old patient with lesion-negative brain MRI. The 3D T1w image acquired at HF is also reported in the last row as gold standard reference

**Fig. 2:**
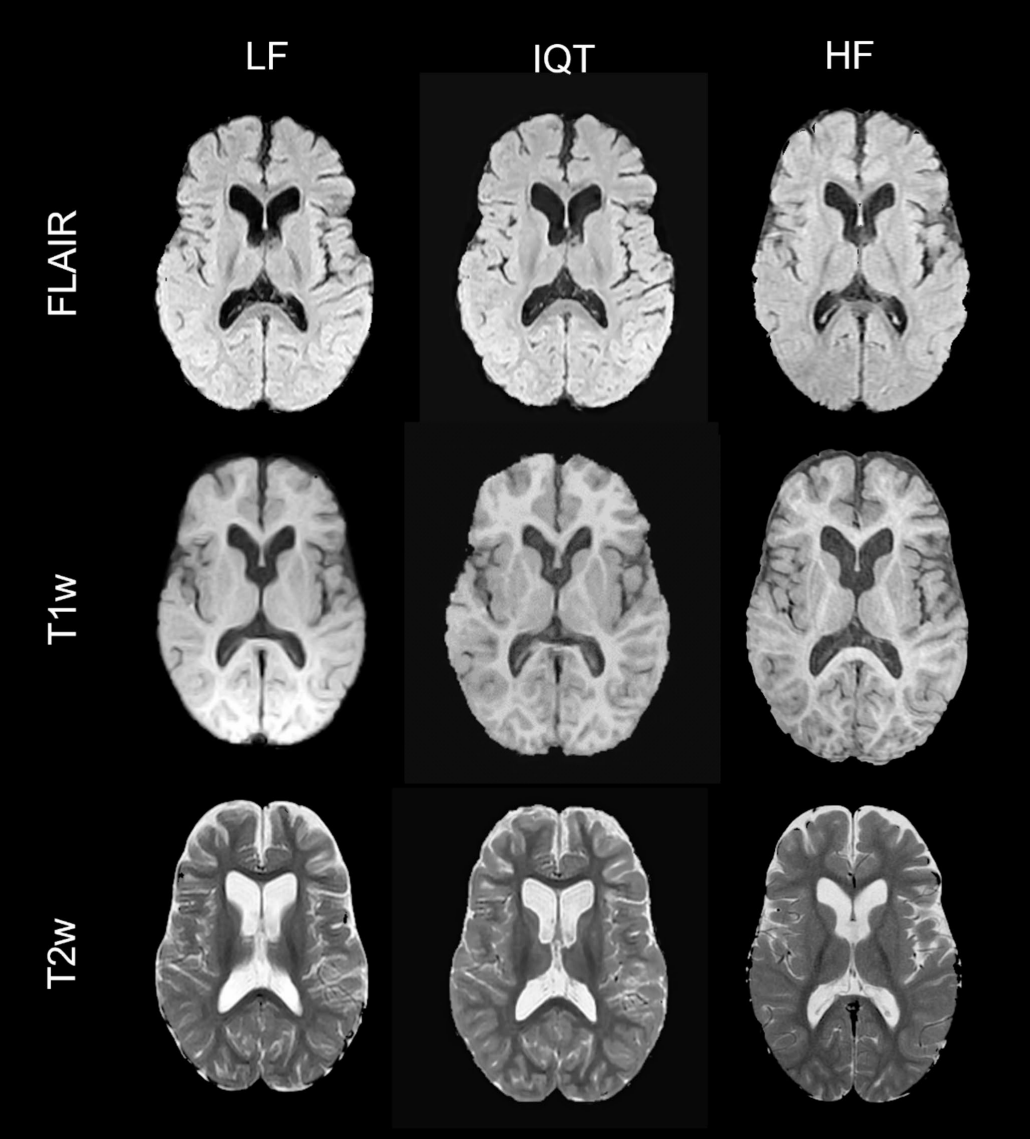
Representative LF, IQT-enhanced and HF axial images for a 12-year-old patient with lesion-negative brain MRI and generalised seizures. On visual inspection, there is an improvement of the grey/white matter differentiation visible in T2w and T1w images

**Fig. 3:**
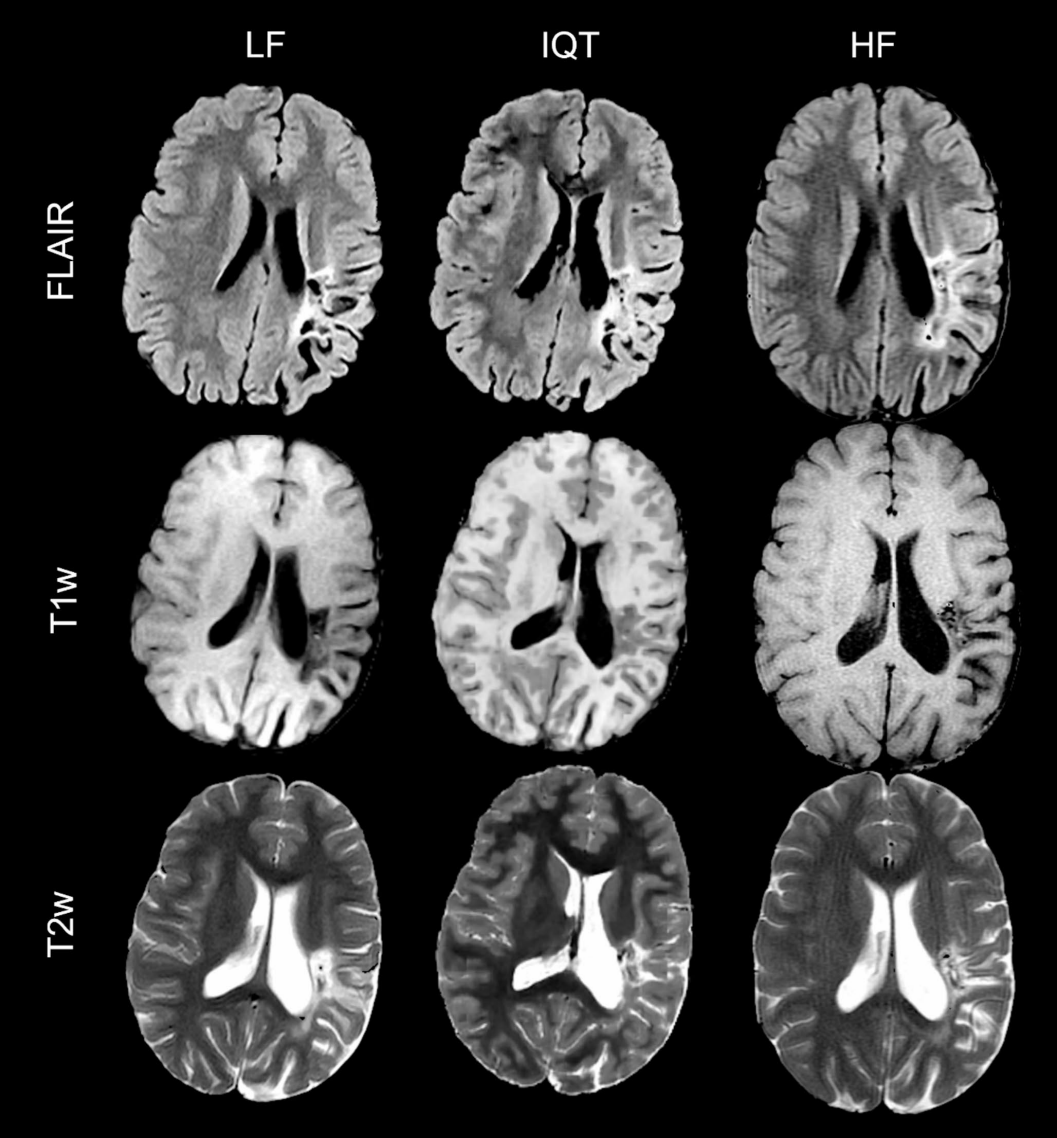
LF, IQT-enhanced and HF images from a 15-year-old patient with long-standing encephalomalacic damage in the middle cerebral artery territory on the left from previous perinatal ischemia. The malacic area is better seen on T2w and FLAIR images, while the partial voluming in IQT on T1w images makes the brain cortex look thick. In this case, the 3D visualisation allowed by IQT, even though advantageous compared to the original 2D images, is not completely accurate and may have negative consequences on lesion identification, which stresses the importance of having multiple sequences

**Fig. 4:**
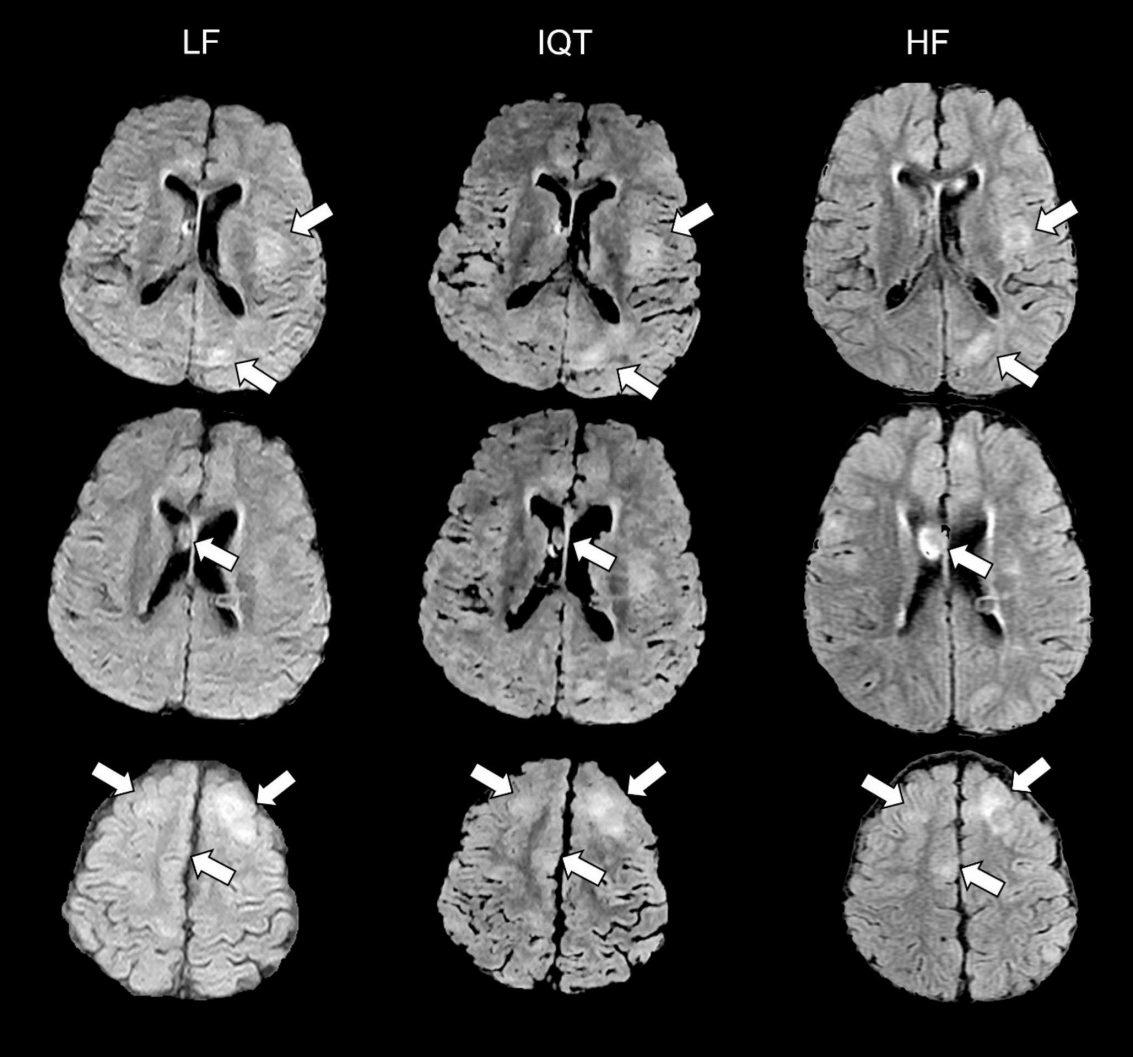
LF, IQT-enhanced and HF images from a 10-year-old patient with tuberous sclerosis. Three different levels are shown in the three rows, with tuberous lesions marked by arrows (cortical dysplasias in the upper and lower rows, sub-ependymal nodule in the middle row). Please note that HF images were acquired in a slightly different orientation than LF images, so the HF slices shown here are as close as possible to the LF and IQT-enhanced ones, but not perfectly matched. The location and extension of the tubers are better appreciated on IQT than on the LF scan. This is critical in epilepsy lesion identification for surgical workup

Visually, IQT substantially improved the image appearance in non-axial planes, as shown by the representative examples of coronal and sagittal reformatted images in Fig. 1. It also slightly improved the contrast between healthy brain tissues, especially in T1w images (Fig. 2). Most of the lesions observed at HF were more clearly visible in IQT-enhanced images than in the corresponding LF images, as shown in Fig. 3 for a patient with encephalomalacia and in Fig. 4 for a patient with tuberous sclerosis.

**Fig. 5:**
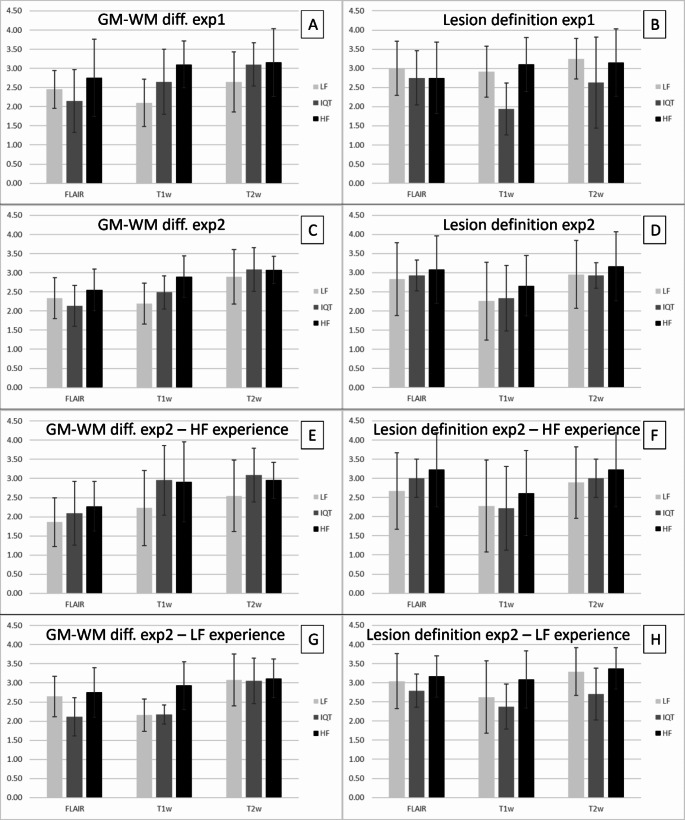
First row: scores from all the reviewers for GM-WM differentiation (**A**) and lesion definition (**B**) when seeing the images independently (experiment 1). Second row: scores from all the reviewers for GM-WM differentiation (**C**) and lesion definition (**D**) when seeing all the images side by side (experiment 2). Third row: scores from the reviewers with specialist expertise in neuroimaging at HF, for GM-WM differentiation (**E**) and lesion definition (**F**), when seeing all the images side by side (experiment 2). Fourth row: scores from the reviewers with general radiology experience mainly at LF, for GM-WM differentiation (**G**) and lesion definition (**H**), when seeing all the images side by side (experiment 2)

In experiment 1, when viewing each set of images independently, reviewers scored the GM-WM differentiation in IQT-enhanced images as higher than LF but lower than HF for T1w and T2w images, with an increase of 26% and 17% respectively with respect to LF. However, the GM-WM differentiation in IQT-enhanced FLAIR images was 12% lower than LF and the lesion definition on all images received lower average scores for IQT than for either LF or HF, with an average difference with respect to LF of 8%, 34% and 19% for FLAIR, T1w and T2w images respectively (Fig. 5, panels A and B).

When the 3 sets of images were evaluated side by side in experiment 2, however, all the lesion definition scores for IQT improved and became 3% higher than LF for FLAIR and T1w images (Fig. 5, panels C and D). The GM-WM differentiation scores had very similar trends to those in experiment 1.

None of the differences in GM-WM differentiation or lesion definition scores between IQT and LF or HF were statistically significant, probably due to the small sample analysed.

We also analysed the scores in experiment 2 separately for the reviewers with expertise in paediatric neuroimaging at HF and those with experience in general radiology at LF. The former group scored the GM-WM differentiation in the IQT-enhanced images as intermediate between LF and HF for FLAIR and even higher than both for T1w and T2w images, and lesion definition in IQT-enhanced images as intermediate between LF and HF for FLAIR and T2w, and slightly lower than both for T1w images. On the other hand, the group with experience in general radiology at LF scored the GM-WM differentiation in IQT-enhanced images as lower than LF and HF for FLAIR, intermediate for T1w and slightly higher than both for T2w images, and the lesion definition on all images as lower than both LF and HF (Fig. 5, panels E-H). This second group of reviewers tended to give higher scores to LF images (7–59% increase on average) and comparatively lower scores to IQT-enhanced images than the first group. All the individual and averaged reviewers scores are available as supplementary material (Supplementary Tables 1 to 11).

## Discussion

In this study we have investigated the enhancement of LF images by IQT from a radiological perspective and showed an increase in contrast and lesion visualisation compared to the original images.

The first paper introducing the IQT implementation for LF MRI showed, both qualitatively and through semi-quantitative radiological evaluations, that IQT can improve the contrast between healthy brain tissues and the spatial resolution, especially in the slice direction, of anatomical MR images (T1w, T2w and FLAIR); in particular, the visualisation of structures in non-axial orientations scored significantly higher for IQT-enhanced than the original LF images [8]. Here we have extended this analysis to both normal and pathological brain tissue and involved a larger group of radiologists with different expertise in paediatric neuroimaging.

Most of the scores given by radiologists to IQT-enhanced images were higher than those of LF images, even though lower than those of the corresponding HF images. This demonstrates that IQT can improve the clinical value of structural brain images acquired at LF, even though the implementation used here does not reach that of actual HF scans.

One exception was the GM-WM differentiation in FLAIR images, which was scored lower than LF on average. This non-ideal performance may be due to the difference in contrast between FLAIR images in the training set and the FLAIR scans used in the experiments. The HCP dataset, arguably the public MRI dataset with the highest quality available, from which we collected our training T1w and T2w images, unfortunately doesn’t include FLAIR. If large collections of high-quality FLAIR images with a more suitable image contrast could be used for training in the future, we expect IQT performance on FLAIR to improve as well.

In contrast with the good performance for GW-WM differentiation, the lesion definition scores of IQT-enhanced images were usually closer to LF than to HF, which means that only a modest, though consistent, improvement could be achieved. One possible explanation for this is that the training set included images of only healthy brains. As it is trained on relatively small patches, IQT learns the local relationship between the intensity of neighbouring voxels in HF images and we assume that this is sufficiently generalisable from healthy to pathological brains at least in the case of epilepsy. Including abnormalities in the training set could be beneficial and it would be probably necessary for diseases that change brain appearance in more drastic ways, e.g. brain tumours. We plan to test this hypothesis in the future; however, it should be noted that introducing abnormalities to training data may increase the likelihood of hallucination of abnormality (introduction of false positives), which must be carefully evaluated and monitored. Extending IQT to different pathologies will thus require several experiments to properly design the training set.

The current approach based on healthy data is more likely to work better on subtle lesions (such as focal cortical dysplasia), where there is more need for IQT enhancement, and worse on more evident lesions, which benefit little from IQT enhancement as suggested by studies comparing lesion detection scores at 1.5T and 3T [13]. In the current study on a limited dataset, we considered any type of epileptogenic lesion (including ischemic infarction, hypoglycaemia, post-traumatic scarring) and many of them may be evident at any field; this may have not allowed us to appreciate the IQT enhancement of subtle lesions. In a future study with a larger population, we aim to perform a focused analysis on focal cortical dysplasia. We also aim to assess the accuracy in lesion detection by comparing the number of detected lesions in LF and IQT images with the ground truth HF, which is probably more clinically relevant in LMICs than evaluating lesion definition and extension.

There was an interesting difference in the assessment of the enhanced images when presented individually (where both GM-WM differentiation and lesion definition scores were lower than LH and HF) than when they were presented together (where they were between LH and HF). One possibility is that the IQT-enhanced images have a less familiar appearance than LF and HF images, so that, when presented individually, they are interpreted with less diagnostic confidence leading to a lower score. When presented with the LF and HF equivalents, the relative quality for lesion visualisation is judged with this reduction in diagnostic confidence mitigated as a factor owing to the ability to cross compare. Similarly, we observed a difference between the scores provided by paediatric neuroradiologists with experience on HF and radiologists with experience mostly on LF MRI, with the latter giving higher scores to LF images and lower scores to IQT-enhanced images. This most likely reflects the different expertise in the two groups. Radiologists who are not used to looking at HF images may need some additional time and training to confidently extract information out of HF and HF-like IQT-enhanced images. In future clinical applications of IQT in LMICs, we can envisage giving all radiologists specific training for this purpose, possibly as part of exchange programs between LMICs and HICs. To make our method easier to deploy in a clinical context, we may also investigate the possibility of augmenting the images in the training set to increase the generalisability of the IQT model and reduce the pre-processing steps needed, especially brain extraction. We will also work on improving the IQT algorithm or post-processing the enhanced images to correct artifacts and features that may look unnatural to radiologists.

Furthermore, in this study we worked on images with thick slices, as routinely acquired at LF, to be able to make meaningful comparisons with the current clinical standard. However, this scenario is particularly challenging for IQT or super-resolution algorithm; in future applications we will test different acquisition protocols to obtain images that may be more suitable as input to IQT even though possibly worse for human evaluation, e.g. with more isotropic spatial resolution, different SNR or contrasts.

This proof-of-concept study focused on overt pathology in a limited number of subjects, with the aim to provide preliminary evidence of the relevance and applicability of IQT for low-field MRI, which is critical in low-resource settings. Once this is assessed, we plan to work on a larger study with more subjects undergoing both LF and HF MRI and IQT-enhanced images reviewed by a group of radiologists with different levels of expertise. With a larger population we expect to be able to analyse different lesion types separately, and to better understand where the current IQT implementation for LF MRI can help most and how it should be improved.

Our current results suggest that IQT could be an important tool to enhance the diagnostic power of low-field MRI and have an impact on radiology practice in low- and middle-income countries, where LF scanners are common and portable systems have recently been introduced to increase access to imaging [14–16]. IQT could also be extended to give a contribution in other clinical applications than those preliminarily investigated here.

## Electronic supplementary material

Below is the link to the electronic supplementary material.

Supplementary Material 1

## Data Availability

The anonymised patient images (raw, post-processed and IQT-enhanced) are available on request. For the training model and data please refer to https://github.com/hongxiangharry/Stochastic-IQT and [8].
