# Supplementary material for: Evaluation of epilepsy lesion visualisation enhancement in low-field MRI using image quality transfer: a preliminary investigation of clinical potential for applications in developing countries
Source: Neuroradiology. 2024 Sep 6;66(12):2243–52. doi: 10.1007/s00234-024-03448-2 (PMC11611997; doi:10.1007/s00234-024-03448-2)
Supplement: Supplementary file 1 — Supplementary Material 1 [file 234_2024_3448_MOESM1_ESM.docx]

**Supplementary table 1**

| **ID** | **GM/WM diff** | | | | | | | | | **lesion definition** | | | | | | | | |
| --- | --- | --- | --- | --- | --- | --- | --- | --- | --- | --- | --- | --- | --- | --- | --- | --- | --- | --- |
|  | **LF** | | | **IQT** | | | **HF** | | | **LF** | | | **IQT** | | | **HF** | | |
|  | **FLAIR** | **T1** | **T2** | **FLAIR** | **T1** | **T2** | **FLAIR** | **T1** | **T2** | **FLAIR** | **T1** | **T2** | **FLAIR** | **T1** | **T2** | **FLAIR** | **T1** | **T2** |
| 1 | 2 | 2 | 2 |  |  |  | 4 | 4 | 4 | 4 | 4 | 4 |  |  |  | 4 | 4 | 4 |
| 2 | 2 | 2 | 2 | 3 | 4 | 4 |  |  |  | N/A | N/A | N/A | N/A | N/A | N/A | N/A | N/A | N/A |
| 3 | 2.5 | 2 | 3.5 | 2 | 2.5 | 3 | 3 | 3 | 3 | 2 | 2 | 3 | 3 | 1 | 1 | 3 | 2 | 3 |
| 4 |  |  |  | 3 | 4 | 4 | 2.5 | 3 | 4 |  |  |  | 4 | 2 | 4 | 3.5 | 3 | 3 |
| 5 | 3 | 3 | 3 | 2 | 2.5 | 3 | 2 | 3 | 3 | 3 | 3 | 3 | 2 | 2.5 | 3 | 3 | 3 | 3 |
| 6 | 3 | 1 | 3 | 2 | 1.5 | 3 | 3.5 | 3.5 | 4 |  |  |  | 2 | 1 | 4 | 1 | 4 | 1 |
| 7 | 3 | 2.5 | 4 | 1.5 | 2 | 3 | 4 | 4 | 4 | 3.5 | 3 | 3.5 | 3 | 3 | 3 | 4 | 4 | 4 |
| 8 | 2 | 1.5 | 1.5 | 1 | 2 | 2 | 1.5 | 2 | 1.5 | 2.5 | 2.5 | 2.5 | 2 | 2 | 2 | 4 | 4 | 3.5 |
| 9 | 2 | 2 | 2 | 3 | 3 | 3 |  |  |  | N/A | N/A | N/A | N/A | N/A | N/A | N/A | N/A | N/A |
| 10 |  |  |  | 1 | 3 | 3 | 3 | 3 | 3 |  |  |  | 3 | 2 | 1 | 3 | 3 | 3 |
| 11 | 2 | 2 | 2.5 | 3 | 2 | 3 | 3 | 2.5 | 3 | 3 | 3 | 3.5 | 3 | 2 | 3 | 3 | 3 | 3 |
| 12 | 3 | 3 | 3 |  |  |  | 1 | 3 | 2 | N/A | N/A | N/A | N/A | N/A | N/A | N/A | N/A | N/A |
| Mean | 2.45 | 2.10 | 2.65 | 2.15 | 2.65 | 3.10 | 2.75 | 3.10 | 3.15 | 3.00 | 2.92 | 3.25 | 2.75 | 1.94 | 2.63 | 3.17 | 3.33 | 3.06 |
| std | 0.50 | 0.61 | 0.78 | 0.82 | 0.85 | 0.57 | 1.01 | 0.61 | 0.88 | 0.71 | 0.66 | 0.52 | 0.71 | 0.68 | 1.19 | 0.94 | 0.71 | 0.88 |

**Supplementary table 1**: Combined scores from all the radiologists for gray/white matter differentiation and lesion definition when seeing the images independently (**experiment 1**). Each radiologist reviewed one set of images (randomly chosen between LF, IQT or HF) from each of 6 subjects.

**Supplementary table 2**

| **ID** | **GM/WM diff** | | | | | | | | | **lesion definition** | | | | | | | | | **Diagnosis on images** |
| --- | --- | --- | --- | --- | --- | --- | --- | --- | --- | --- | --- | --- | --- | --- | --- | --- | --- | --- | --- |
|  | **LF** | | | **IQT** | | | **HF** | | | **LF** | | | **IQT** | | | **HF** | | |  |
|  | **FLAIR** | **T1** | **T2** | **FLAIR** | **T1** | **T2** | **FLAIR** | **T1** | **T2** | **FLAIR** | **T1** | **T2** | **FLAIR** | **T1** | **T2** | **FLAIR** | **T1** | **T2** |  |
| 1 | 4 | 4 | 4 | 3 | 3 | 3 | 1 | 1 | 1 | 4 | 4 | 4 | 3 | 3 | 3 | 1 | 1 | 1 | Encephalomalacia |
| 2 | 3 | 3 | 3 | 3 | 3 | 4 | 4 | 4 | 4 | 2 | 3 | 1 | 2 | 1 | 3 | 1 | 1 | 1 | Normal? Left frontal heterotopia. Posterior atrophy |
| 3 |  |  |  |  |  |  |  |  |  |  |  |  |  |  |  |  |  |  |  |
| 4 | 3 | 2 | 3 | 2 | 2 | 2 | 3 | 3 | 4 | 3 | 3 | 3 | 2 | 2 | 2 | 4 | 4 | 4 | Thalamic hyperintensity. Old occipital stroke. |
| 5 |  |  |  |  |  |  |  |  |  |  |  |  |  |  |  |  |  |  |  |
| 6 |  |  |  |  |  |  |  |  |  |  |  |  |  |  |  |  |  |  |  |
| 7 |  |  |  |  |  |  |  |  |  |  |  |  |  |  |  |  |  |  |  |
| 8 |  |  |  |  |  |  |  |  |  |  |  |  |  |  |  |  |  |  |  |
| 9 | 4 | 4 | 4 | 3 | 2 | 3 |  |  |  |  |  |  | 4 | 4 | 4 |  |  |  |  |
| 10 | 2 | 2 | 4 | 4 | 4 | 4 | 3 | 1 | 4 | 2 | 2 | 4 | 3 | 1 | 3 | 4 | 2 | 3 | Multiple cortical hyperintensities |
| 11 |  |  |  |  |  |  |  |  |  |  |  |  |  |  |  |  |  |  |  |
| 12 | 4 | 3 | 4 | 2 | 2 | 3 | 4 | 4 | 4 | 3 | 3 | 3 | 3 | 3 | 4 | 4 | 4 | 4 | Mesial temporal lobe atrophy |

**Supplementary table 2:** Scores from radiologist #**1** for gray/white matter differentiation and lesion definition when seeing all the images side by side (experiment 2).

**Supplementary table 3**

| **ID** | **GM/WM diff** | | | | | | | | | **lesion definition** | | | | | | | | | **Diagnosis on images** |
| --- | --- | --- | --- | --- | --- | --- | --- | --- | --- | --- | --- | --- | --- | --- | --- | --- | --- | --- | --- |
|  | **LF** | | | **IQT** | | | **HF** | | | **LF** | | | **IQT** | | | **HF** | | |  |
|  | **FLAIR** | **T1** | **T2** | **FLAIR** | **T1** | **T2** | **FLAIR** | **T1** | **T2** | **FLAIR** | **T1** | **T2** | **FLAIR** | **T1** | **T2** | **FLAIR** | **T1** | **T2** |  |
| 1 |  |  |  |  |  |  |  |  |  |  |  |  |  |  |  |  |  |  |  |
| 2 |  |  |  |  |  |  |  |  |  |  |  |  |  |  |  |  |  |  |  |
| 3 | 1 | 2 | 2 | 1 | 3 | 3 | 3 | 4 | 4 | 4 | 1 | 3 | 3 | 1 | 3 | 4 | 2 | 4 | Occipital cortico-subcortical lesions (neonatal hypoglycaemia) |
| 4 | 2 | 4 | 4 | 1 | 3 | 3 | 2 | 1 | 2 | 3 | 4 | 4 | 3 | 1 | 3 | 4 | 1 | 3 | Right PCA infarct |
| 5 |  |  |  |  |  |  |  |  |  |  |  |  |  |  |  |  |  |  |  |
| 6 | 2 | 1 | 2 | 2 | 4 | 3 | 2 | 3 | 3 | 1 | 1 | 1 | 3 | 3 | 3 | 1 | 1 | 1 | Left temporal pole blurring FCD |
| 7 | 3 | 4 | 4 | 2 | 1 | 2 | 3 | 2 | 3 | 4 | 4 | 4 | 4 | 4 | 4 | 3 | 3 | 3 | Left brain tumour |
| 8 | 1 | 1 | 1 | 1 | 3 | 2 | 2 | 4 | 3 | 3 | 3 | 3 | 3 | 3 | 3 | 4 | 4 | 4 | Hydrocephalus |
| 9 |  |  |  |  |  |  |  |  |  |  |  |  |  |  |  |  |  |  |  |
| 10 |  |  |  |  |  |  |  |  |  |  |  |  |  |  |  |  |  |  |  |
| 11 | 2 | 3 | 3 | 3 | 2 | 2 | 1 | 2 | 2 | 3 | 3 | 3 | 3 | 3 | 3 | 3 | 3 | 3 | Gliomalacic changes |
| 12 |  |  |  |  |  |  |  |  |  |  |  |  |  |  |  |  |  |  |  |

**Supplementary table 3:** Scores from radiologist #**2** for gray/white matter differentiation and lesion definition when seeing all the images side by side (experiment 2).

**Supplementary table 4**

| **ID** | **GM/WM diff** | | | | | | | | | **lesion definition** | | | | | | | | | **Diagnosis on images** |
| --- | --- | --- | --- | --- | --- | --- | --- | --- | --- | --- | --- | --- | --- | --- | --- | --- | --- | --- | --- |
|  | **LF** | | | **IQT** | | | **HF** | | | **LF** | | | **IQT** | | | **HF** | | |  |
|  | **FLAIR** | **T1** | **T2** | **FLAIR** | **T1** | **T2** | **FLAIR** | **T1** | **T2** | **FLAIR** | **T1** | **T2** | **FLAIR** | **T1** | **T2** | **FLAIR** | **T1** | **T2** |  |
| 1 | 3 | 2 | 3 | 3 | 3 | 4 | 3 | 4 | 4 | 4 | 3 | 4 | 4 | 3 | 4 | 4 | 4 | 4 | Left parietal lobe chronic infarct( MCA territory) |
| 2 | 3 | 2 | 4 | 2 | 2 | 3 | 4 | 3 | 4 | 1 | 1 | 1 | 1 | 1 | 1 | 1 | 1 | 1 | No obvious abnormality |
| 3 |  |  |  |  |  |  |  |  |  |  |  |  |  |  |  |  |  |  |  |
| 4 | 4 | 2 | 4 | 3 | 4 | 4 | 2 | 3 | 4 | 4 | 3 | 4 | 3 | 3 | 4 | 4 | 4 | 4 | Right occipital lobe chronic encephalomalacia? Chronic infarct |
| 5 |  |  |  |  |  |  |  |  |  |  |  |  |  |  |  |  |  |  |  |
| 6 |  |  |  |  |  |  |  |  |  |  |  |  |  |  |  |  |  |  |  |
| 7 |  |  |  |  |  |  |  |  |  |  |  |  |  |  |  |  |  |  |  |
| 8 |  |  |  |  |  |  |  |  |  |  |  |  |  |  |  |  |  |  |  |
| 9 | 3 | 2 | 4 | 3 | 2 | 4 | 3 | 2 | 4 | 1 | 1 | 1 | 1 | 1 | 1 | 1 | 1 | 1 | No obvious abnormality |
| 10 | 3 | 1 | 3 | 3 | 2 | 4 | 3 | 4 | 4 | 3 | 1 | 2 | 3 | 2 | 2 | 4 | 3 | 3 | Sub-ependymal hamartomas (tuberous sclerosis) |
| 11 |  |  |  |  |  |  |  |  |  |  |  |  |  |  |  |  |  |  |  |
| 12 | 3 | 2 | 4 | 2 | 4 | 4 | 3 | 4 | 4 | 4 | 3 | 3 | 4 | 4 | 3 | 4 | 4 | 4 | Mild to moderate cerebral atrophy |

**Supplementary table 4:** Scores from radiologist #**3** for gray/white matter differentiation and lesion definition when seeing all the images side by side (experiment 2).

**Supplementary table 5**

| **ID** | **GM/WM diff** | | | | | | | | | **lesion definition** | | | | | | | | | **Diagnosis on images** |
| --- | --- | --- | --- | --- | --- | --- | --- | --- | --- | --- | --- | --- | --- | --- | --- | --- | --- | --- | --- |
|  | **LF** | | | **IQT** | | | **HF** | | | **LF** | | | **IQT** | | | **HF** | | |  |
|  | **FLAIR** | **T1** | **T2** | **FLAIR** | **T1** | **T2** | **FLAIR** | **T1** | **T2** | **FLAIR** | **T1** | **T2** | **FLAIR** | **T1** | **T2** | **FLAIR** | **T1** | **T2** |  |
| 1 | 2 | 2 | 2 | 3 | 4 | 4 | 2 | 3 | 3 | 2 | 2 | 3 | 3 | 2 | 3 | 3 | 4 | 4 | Left MCA ischemia |
| 2 |  |  |  |  |  |  |  |  |  |  |  |  |  |  |  |  |  |  |  |
| 3 |  |  |  |  |  |  |  |  |  |  |  |  |  |  |  |  |  |  |  |
| 4 |  |  |  |  |  |  |  |  |  |  |  |  |  |  |  |  |  |  |  |
| 5 | 1 | 2 | 2 | 2 | 3 | 4 | 2 | 3 | 3 | 2 | 2 | 3 | 2 | 2 | 3 | 3 | 3 | 4 | Hypoxic ischemic injury |
| 6 |  |  |  |  |  |  |  |  |  |  |  |  |  |  |  |  |  |  |  |
| 7 |  |  |  |  |  |  |  |  |  |  |  |  |  |  |  |  |  |  |  |
| 8 |  |  |  |  |  |  |  |  |  |  |  |  |  |  |  |  |  |  |  |
| 9 | 2 | 2 | 3 | 3 | 3 | 4 | 3 | 2 | 3 | N/A | N/A | N/A | N/A | N/A | N/A | N/A | N/A | N/A | N/A |
| 10 | 2 | 2 | 3 | 3 | 2 | 3 | 3 | 4 | 3 | 2 | 1 | 2 | 3 | 1 | 2 | 4 | 3 | 3 | Tuberous sclerosis (sub ependymal nodules and tubers) |
| 11 | 3 | 2 | 3 | 3 | 3 | 4 | 3 | 2 | 3 | 3 | 2 | 3 | 3 | 3 | 3 | 3 | 2 | 3 | Left MCA ischemia |
| 12 | 2 | 2 | 2 | 2 | 4 | 3 | 1 | 4 | 3 | N/A | N/A | N/A | N/A | N/A | N/A | N/A | N/A | N/A | N/A |

**Supplementary table 5:** Scores from radiologist #**4** for gray/white matter differentiation and lesion definition when seeing all the images side by side (experiment 2).

**Supplementary table 6**

| **ID** | **GM/WM diff** | | | | | | | | | **lesion definition** | | | | | | | | | **Diagnosis on images** |
| --- | --- | --- | --- | --- | --- | --- | --- | --- | --- | --- | --- | --- | --- | --- | --- | --- | --- | --- | --- |
|  | **LF** | | | **IQT** | | | **HF** | | | **LF** | | | **IQT** | | | **HF** | | |  |
|  | **FLAIR** | **T1** | **T2** | **FLAIR** | **T1** | **T2** | **FLAIR** | **T1** | **T2** | **FLAIR** | **T1** | **T2** | **FLAIR** | **T1** | **T2** | **FLAIR** | **T1** | **T2** |  |
| 1 | 2 | 1 | 3 | 2 | 1 | 3 | 2 | 3 | 4 | 3 | 3 | 4 | 3 | 2 | 4 | 3 | 3 | 4 | Encephalomalacia? Following subdural haematoma |
| 2 | 3 | 2 | 4 | 2 | 2 | 4 | 2 | 3 | 4 | 3 | 2 | 3 | 3 | 2 | 3 | 3 | 3 | 3 | Chronic water shed atrophy (Injury) |
| 3 |  |  |  |  |  |  |  |  |  |  |  |  |  |  |  |  |  |  |  |
| 4 |  |  |  |  |  |  |  |  |  |  |  |  |  |  |  |  |  |  |  |
| 5 | 1 | 2 | 2 | 1 | 2 | 3 | 1 | 2 | 2 | 2 | 2 | 2 | 2 | 2 | 2 | 3 | 2 | 4 | Post cerebral encephalomalacia w/ ventriculomegaly. FCD w/ balloon cells |
| 6 |  |  |  |  |  |  |  |  |  |  |  |  |  |  |  |  |  |  |  |
| 7 |  |  |  |  |  |  |  |  |  |  |  |  |  |  |  |  |  |  |  |
| 8 |  |  |  |  |  |  |  |  |  |  |  |  |  |  |  |  |  |  |  |
| 9 | 3 | 2 | 4 | 3 | 2 | 4 | 3 | 3 | 4 | NA | NA | NA | NA | NA | NA | NA | NA | NA | Normal study |
| 10 | 2 | 2 | 3 | 1 | 1 | 3 | 3 | 3 | 2 | 3 | 1 | 2 | 3 | 1 | 2 | 3 | 1 | 2 | Multifocal hyperintensities probably due to Hypoxic ischemia |
| 11 |  |  |  |  |  |  |  |  |  |  |  |  |  |  |  |  |  |  |  |
| 12 | 2 | 3 | 4 | 2 | 3 | 4 | 2 | 4 | 3 | 2 | 3 | 4 | 3 | 3 | 3 | 3 | 3 | 4 | Widening of the entire CSF spaces. Generalised mild cerebral atrophy. |

**Supplementary table 6:** Scores from radiologist #**5** for gray/white matter differentiation and lesion definition when seeing all the images side by side (experiment 2).

**Supplementary table 7**

| **ID** | **GM/WM diff** | | | | | | | | | **lesion definition** | | | | | | | | | **Diagnosis on images** |
| --- | --- | --- | --- | --- | --- | --- | --- | --- | --- | --- | --- | --- | --- | --- | --- | --- | --- | --- | --- |
|  | **LF** | | | **IQT** | | | **HF** | | | **LF** | | | **IQT** | | | **HF** | | |  |
|  | **FLAIR** | **T1** | **T2** | **FLAIR** | **T1** | **T2** | **FLAIR** | **T1** | **T2** | **FLAIR** | **T1** | **T2** | **FLAIR** | **T1** | **T2** | **FLAIR** | **T1** | **T2** |  |
| 1 |  |  |  |  |  |  |  |  |  |  |  |  |  |  |  |  |  |  |  |
| 2 | 3 | 3 | 3 | 2 | 2 | 3 | 3 | 3 | 3 | N/A | N/A | N/A | N/A | N/A | N/A | N/A | N/A | N/A | Normal |
| 3 | 2 | 2 | 2 | 2 | 2 | 3 | 3 | 3 | 3 | N/A | N/A | N/A | N/A | N/A | N/A | N/A | N/A | N/A | Normal |
| 4 | 3 | 1 | 4 | 3 | 2 | 3 | 3 | 4 | 3 | 3 | 1 | 4 | 3 | 2 | 3 | 3 | 4 | 3 | Infarct |
| 5 |  |  |  |  |  |  |  |  |  |  |  |  |  |  |  |  |  |  |  |
| 6 | 3 | 2 | 3 | 2 | 2 | 3 | 3 | 4 | 3 | N/A | N/A | N/A | N/A | N/A | N/A | N/A | N/A | N/A | Normal |
| 7 | 3 | 3 | 3 | 2 | 2 | 2 | 4 | 3 | 3 | 4 | 4 | 4 | 3 | 3 | 2 | 4 | 4 | 4 | Pilocytic astrocytoma |
| 8 | 3 | 2 | 2 | 2 | 2 | 2 | 2 | 2 | 2 | 3 | 3 | 3 | 3 | 3 | 3 | 3 | 3 | 3 | Callosal agenesis and ventriculomegaly |
| 9 |  |  |  |  |  |  |  |  |  |  |  |  |  |  |  |  |  |  |  |
| 10 |  |  |  |  |  |  |  |  |  |  |  |  |  |  |  |  |  |  |  |
| 11 |  |  |  |  |  |  |  |  |  |  |  |  |  |  |  |  |  |  |  |
| 12 |  |  |  |  |  |  |  |  |  |  |  |  |  |  |  |  |  |  |  |

**Supplementary table 7:** Scores from radiologist #**6** for gray/white matter differentiation and lesion definition when seeing all the images side by side (experiment 2).

**Supplementary table 8**

| **ID** | **GM/WM diff** | | | | | | | | | **lesion definition** | | | | | | | | | **Diagnosis on images** |
| --- | --- | --- | --- | --- | --- | --- | --- | --- | --- | --- | --- | --- | --- | --- | --- | --- | --- | --- | --- |
|  | **LF** | | | **IQT** | | | **HF** | | | **LF** | | | **IQT** | | | **HF** | | |  |
|  | **FLAIR** | **T1** | **T2** | **FLAIR** | **T1** | **T2** | **FLAIR** | **T1** | **T2** | **FLAIR** | **T1** | **T2** | **FLAIR** | **T1** | **T2** | **FLAIR** | **T1** | **T2** |  |
| 1 | 2 | 1 | 3 | 2 | 2 | 3 | 3 | 3 | 4 | 3 | 3 | 3 | 3 | 2 | 4 | 3 | 3 | 3 | Chronic infarct of the left parietal and occipital lobes. |
| 2 | 2 | 2 | 3 | 1 | 2 | 3 | 2 | 2 | 3 | 2 | 1 | 3 | 1 | 2 | 2 | 3 | 2 | 3 | Cerebral aerocele. |
| 3 |  |  |  |  |  |  |  |  |  |  |  |  |  |  |  |  |  |  |  |
| 4 |  |  |  |  |  |  |  |  |  |  |  |  |  |  |  |  |  |  |  |
| 5 | 2 | 2 | 3 | 1 | 2 | 3 | 2 | 3 | 3 | 2 | 2 | 3 | 2 | 2 | 2 | 2 | 3 | 4 | Chronic infarct of Lt par, Lt occ and Rt par lobe. Chronic sinusitis. |
| 6 |  |  |  |  |  |  |  |  |  |  |  |  |  |  |  |  |  |  |  |
| 7 |  |  |  |  |  |  |  |  |  |  |  |  |  |  |  |  |  |  |  |
| 8 |  |  |  |  |  |  |  |  |  |  |  |  |  |  |  |  |  |  |  |
| 9 | 2 | 2 | 4 | 2 | 2 | 4 | 3 | 2 | 3 | 1 | 1 | 1 | 1 | 1 | 1 | 1 | 1 | 1 | Non specific |
| 10 | 2 | 2 | 3 | 2 | 2 | 3 | 3 | 2 | 3 | 2 | 2 | 3 | 2 | 2 | 3 | 2 | 3 | 3 | Choroid plexus papilloma |
| 11 |  |  |  |  |  |  |  |  |  |  |  |  |  |  |  |  |  |  |  |
| 12 | 1 | 2 | 3 | 1 | 2 | 4 | 2 | 3 | 3 | 3 | 3 | 2 | 3 | 4 | 3 | 3 | 3 | 3 | Moderate generalised cerebral atrophy. |

**Supplementary table 8:** Scores from radiologist #**7** for gray/white matter differentiation and lesion definition when seeing all the images side by side (experiment 2).

**Supplementary table 9**

| **ID** | **GM/WM diff** | | | | | | | | | **lesion definition** | | | | | | | | |
| --- | --- | --- | --- | --- | --- | --- | --- | --- | --- | --- | --- | --- | --- | --- | --- | --- | --- | --- |
|  | **LF** | | | **IQT** | | | **HF** | | | **LF** | | | **IQT** | | | **HF** | | |
|  | **FLAIR** | **T1** | **T2** | **FLAIR** | **T1** | **T2** | **FLAIR** | **T1** | **T2** | **FLAIR** | **T1** | **T2** | **FLAIR** | **T1** | **T2** | **FLAIR** | **T1** | **T2** |
| 1 | 2.50 | 2.00 | 2.83 | 2.67 | 2.83 | 3.50 | 2.17 | 2.83 | 3.17 | 3.00 | 2.83 | 3.50 | 3.17 | 2.33 | 3.50 | 2.83 | 3.17 | 3.33 |
| 2 | 2.83 | 2.50 | 3.33 | 2.00 | 2.17 | 3.33 | 3.00 | 3.00 | 3.50 | N/A | N/A | N/A | N/A | N/A | N/A | N/A | N/A | N/A |
| 3 | 1.50 | 2.00 | 2.00 | 1.50 | 2.50 | 3.00 | 3.00 | 3.50 | 3.50 | 4.00 | 1.00 | 3.00 | 3.00 | 1.00 | 3.00 | 4.00 | 2.00 | 4.00 |
| 4 | 2.83 | 2.33 | 3.83 | 2.17 | 2.67 | 3.00 | 2.50 | 2.67 | 3.00 | 3.17 | 2.67 | 3.83 | 2.83 | 1.83 | 3.00 | 3.67 | 3.00 | 3.33 |
| 5 | 1.25 | 2.00 | 2.25 | 1.50 | 2.50 | 3.50 | 1.75 | 2.75 | 2.75 | 2.00 | 2.00 | 2.75 | 2.00 | 2.00 | 2.50 | 2.75 | 2.75 | 4.00 |
| 6 | 2.50 | 1.50 | 2.50 | 2.00 | 3.00 | 3.00 | 2.50 | 3.50 | 3.00 | 1.00 | 1.00 | 1.00 | 3.00 | 3.00 | 3.00 | 1.00 | 1.00 | 1.00 |
| 7 | 3.00 | 3.50 | 3.50 | 2.00 | 1.50 | 2.00 | 3.50 | 2.50 | 3.00 | 4.00 | 4.00 | 4.00 | 3.50 | 3.50 | 3.00 | 3.50 | 3.50 | 3.50 |
| 8 | 2.00 | 1.50 | 1.50 | 1.50 | 2.50 | 2.00 | 2.00 | 3.00 | 2.50 | 3.00 | 3.00 | 3.00 | 3.00 | 3.00 | 3.00 | 3.50 | 3.50 | 3.50 |
| 9 | 2.67 | 2.33 | 3.67 | 2.83 | 2.33 | 3.83 | 3.00 | 2.17 | 3.50 | N/A | N/A | N/A | N/A | N/A | N/A | N/A | N/A | N/A |
| 10 | 2.17 | 1.83 | 3.17 | 2.67 | 2.17 | 3.33 | 3.00 | 3.00 | 3.17 | 2.33 | 1.33 | 2.50 | 2.83 | 1.33 | 2.33 | 3.50 | 2.50 | 2.83 |
| 11 | 2.50 | 2.50 | 3.00 | 3.00 | 2.50 | 3.00 | 2.00 | 2.00 | 2.50 | 3.00 | 2.50 | 3.00 | 3.00 | 3.00 | 3.00 | 3.00 | 2.50 | 3.00 |
| 12 | 2.33 | 2.33 | 3.17 | 1.83 | 3.17 | 3.50 | 2.17 | 3.83 | 3.33 | N/A | N/A | N/A | N/A | N/A | N/A | N/A | N/A | N/A |
| Mean | 2.34 | 2.19 | 2.90 | 2.14 | 2.49 | 3.08 | 2.55 | 2.90 | 3.08 | 2.83 | 2.26 | 2.95 | 2.93 | 2.33 | 2.93 | 3.08 | 2.66 | 3.17 |
| std | 0.53 | 0.54 | 0.71 | 0.54 | 0.43 | 0.57 | 0.54 | 0.54 | 0.36 | 0.95 | 1.01 | 0.88 | 0.40 | 0.85 | 0.33 | 0.88 | 0.79 | 0.90 |

**Supplementary table 9:** Average scores across all radiologists for gray/white matter differentiation and lesion definition when seeing all the images side by side (**experiment 2**).

**Supplementary table 10**

| **ID** | **GM/WM diff** | | | | | | | | | **lesion definition** | | | | | | | | |
| --- | --- | --- | --- | --- | --- | --- | --- | --- | --- | --- | --- | --- | --- | --- | --- | --- | --- | --- |
|  | **LF** | | | **IQT** | | | **HF** | | | **LF** | | | **IQT** | | | **HF** | | |
|  | **FLAIR** | **T1** | **T2** | **FLAIR** | **T1** | **T2** | **FLAIR** | **T1** | **T2** | **FLAIR** | **T1** | **T2** | **FLAIR** | **T1** | **T2** | **FLAIR** | **T1** | **T2** |
| 1 | 2 | 2 | 2 | 3 | 4 | 4 | 2 | 3 | 3 | 2 | 2 | 3 | 3 | 2 | 3 | 3 | 4 | 4 |
| 2 |  |  |  |  |  |  |  |  |  | N/A | N/A | N/A | N/A | N/A | N/A | N/A | N/A | N/A |
| 3 | 1 | 2 | 2 | 1 | 3 | 3 | 3 | 4 | 4 | 4 | 1 | 3 | 3 | 1 | 3 | 4 | 2 | 4 |
| 4 | 2 | 4 | 4 | 1 | 3 | 3 | 2 | 1 | 2 | 3 | 4 | 4 | 3 | 1 | 3 | 4 | 1 | 3 |
| 5 | 1 | 2 | 2 | 2 | 3 | 4 | 2 | 3 | 3 | 2 | 2 | 3 | 2 | 2 | 3 | 3 | 3 | 4 |
| 6 | 2 | 1 | 2 | 2 | 4 | 3 | 2 | 3 | 3 | 1 | 1 | 1 | 3 | 3 | 3 | 1 | 1 | 1 |
| 7 | 3 | 4 | 4 | 2 | 1 | 2 | 3 | 2 | 3 | 4 | 4 | 4 | 4 | 4 | 4 | 3 | 3 | 3 |
| 8 | 1 | 1 | 1 | 1 | 3 | 2 | 2 | 4 | 3 | 3 | 3 | 3 | 3 | 3 | 3 | 4 | 4 | 4 |
| 9 | 2 | 2 | 3 | 3 | 3 | 4 | 3 | 2 | 3 | N/A | N/A | N/A | N/A | N/A | N/A | N/A | N/A | N/A |
| 10 | 2 | 2 | 3 | 3 | 2 | 3 | 3 | 4 | 3 | 2 | 1 | 2 | 3 | 1 | 2 | 4 | 3 | 3 |
| 11 | 2.5 | 2.5 | 3 | 3 | 2.5 | 3 | 2 | 2 | 2.5 | 3 | 2.5 | 3 | 3 | 3 | 3 | 3 | 2.5 | 3 |
| 12 | 2 | 2 | 2 | 2 | 4 | 3 | 1 | 4 | 3 | N/A | N/A | N/A | N/A | N/A | N/A | N/A | N/A | N/A |
| Mean | 1.86 | 2.23 | 2.55 | 2.09 | 2.95 | 3.09 | 2.27 | 2.91 | 2.95 | 2.67 | 2.28 | 2.89 | 3.00 | 2.22 | 3.00 | 3.22 | 2.61 | 3.22 |
| std | 0.64 | 0.98 | 0.93 | 0.83 | 0.91 | 0.70 | 0.65 | 1.04 | 0.47 | 1.00 | 1.20 | 0.93 | 0.50 | 1.09 | 0.50 | 0.97 | 1.11 | 0.97 |

**Supplementary table 10:** Average scores across neuroradiologists with experience at **high field** for gray/white matter differentiation and lesion definition when seeing all the images side by side (experiment 2).

**Supplementary table 11**

| **ID** | **GM/WM diff** | | | | | | | | | **lesion definition** | | | | | | | | |
| --- | --- | --- | --- | --- | --- | --- | --- | --- | --- | --- | --- | --- | --- | --- | --- | --- | --- | --- |
|  | **LF** | | | **IQT** | | | **HF** | | | **LF** | | | **IQT** | | | **HF** | | |
|  | **FLAIR** | **T1** | **T2** | **FLAIR** | **T1** | **T2** | **FLAIR** | **T1** | **T2** | **FLAIR** | **T1** | **T2** | **FLAIR** | **T1** | **T2** | **FLAIR** | **T1** | **T2** |
| 1 | 2.75 | 2.00 | 3.25 | 2.50 | 2.25 | 3.25 | 2.25 | 2.75 | 3.25 | 3.50 | 3.25 | 3.75 | 3.25 | 2.50 | 3.75 | 2.75 | 2.75 | 3.00 |
| 2 | 2.83 | 2.50 | 3.33 | 2.00 | 2.17 | 3.33 | 3.00 | 3.00 | 3.50 | N/A | N/A | N/A | N/A | N/A | N/A | N/A | N/A | N/A |
| 3 | 2.00 | 2.00 | 2.00 | 2.00 | 2.00 | 3.00 | 3.00 | 3.00 | 3.00 |  |  |  |  |  |  |  |  |  |
| 4 | 3.25 | 1.50 | 3.75 | 2.75 | 2.50 | 3.00 | 2.75 | 3.50 | 3.50 | 3.25 | 2.00 | 3.75 | 2.75 | 2.25 | 3.00 | 3.50 | 4.00 | 3.50 |
| 5 | 1.50 | 2.00 | 2.50 | 1.00 | 2.00 | 3.00 | 1.50 | 2.50 | 2.50 | 2.00 | 2.00 | 2.50 | 2.00 | 2.00 | 2.00 | 2.50 | 2.50 | 4.00 |
| 6 | 3.00 | 2.00 | 3.00 | 2.00 | 2.00 | 3.00 | 3.00 | 4.00 | 3.00 |  |  |  |  |  |  |  |  |  |
| 7 | 3.00 | 3.00 | 3.00 | 2.00 | 2.00 | 2.00 | 4.00 | 3.00 | 3.00 | 4.00 | 4.00 | 4.00 | 3.00 | 3.00 | 2.00 | 4.00 | 4.00 | 4.00 |
| 8 | 3.00 | 2.00 | 2.00 | 2.00 | 2.00 | 2.00 | 2.00 | 2.00 | 2.00 | 3.00 | 3.00 | 3.00 | 3.00 | 3.00 | 3.00 | 3.00 | 3.00 | 3.00 |
| 9 | 3.00 | 2.50 | 4.00 | 2.75 | 2.00 | 3.75 | 3.00 | 2.25 | 3.75 | N/A | N/A | N/A | N/A | N/A | N/A | N/A | N/A | N/A |
| 10 | 2.25 | 1.75 | 3.25 | 2.50 | 2.25 | 3.50 | 3.00 | 2.50 | 3.25 | 2.50 | 1.50 | 2.75 | 2.75 | 1.50 | 2.50 | 3.25 | 2.25 | 2.75 |
| 11 |  |  |  |  |  |  |  |  |  |  |  |  |  |  |  |  |  |  |
| 12 | 2.50 | 2.50 | 3.75 | 1.75 | 2.75 | 3.75 | 2.75 | 3.75 | 3.50 | N/A | N/A | N/A | N/A | N/A | N/A | N/A | N/A | N/A |
| Mean | 2.64 | 2.16 | 3.08 | 2.11 | 2.17 | 3.05 | 2.75 | 2.93 | 3.11 | 3.04 | 2.63 | 3.29 | 2.79 | 2.38 | 2.71 | 3.17 | 3.08 | 3.38 |
| std | 0.53 | 0.42 | 0.67 | 0.50 | 0.25 | 0.59 | 0.65 | 0.62 | 0.50 | 0.71 | 0.95 | 0.62 | 0.43 | 0.59 | 0.68 | 0.54 | 0.75 | 0.54 |

**Supplementary table 11:** Average scores across radiologists with experience at **low field**, for gray/white matter differentiation and lesion definition when seeing all the images side by side (experiment 2).
